# Alternative Polyadenylation Contributes to Fibroblast Senescence in Pulmonary Fibrosis

**DOI:** 10.1111/acel.70179

**Published:** 2025-07-30

**Authors:** Jingjing Huang, Maria Jose Gacha‐Garay, Yu Wang, Scott D. Collum, Rene A. Girard, Hydia Puente, Seung‐Hee Yoo, Rahat Hussain, Jayeshkumar Patel, Manish Patel, Eric J. Wagner, Hari KrishnaYalamanchili, Harry Karmouty‐Quintana, Zheng Chen, Tingting Mills

**Affiliations:** ^1^ Department of Biochemistry and Molecular Biology The University of Texas Health Science Center at Houston Houston Texas USA; ^2^ Department of Geriatrics The Fourth Affiliated Hospital of Nanjing Medical University Nanjing China; ^3^ The Center for Advanced Cardiopulmonary Therapies and Transplantation University of Texas Health Science Center at Houston Houston Texas USA; ^4^ Department of Biochemistry and Biophysics University of Rochester Rochester New York USA; ^5^ Department of Pediatrics Neurology Baylor College of Medicine Houston Texas USA; ^6^ Division of Pulmonary, Critical Care and Sleep Medicine, Department of Internal Medicine, McGovern Medical School University of Texas Health Science Center at Houston Houston Texas USA

**Keywords:** alternative polyadenylation, fibroblast, NUDT21, pulmonary fibrosis, senescence

## Abstract

Idiopathic pulmonary fibrosis (IPF) is a prevalent and deadly age‐related disease characterized by chronic, progressive, and irreversible fibrosis. A key effector cell population in the fibroproliferative response is the fibroblasts. Fibroblast cell senescence gradually worsens during aging, and the acquisition of a senescence‐associated secretory phenotype (SASP) turns senescent fibroblasts into pro‐inflammatory cells. However, the mechanism promoting senescence in IPF, especially at the post‐transcriptional level, is poorly understood. We recently discovered that Nudix Hydrolase 21 (NUDT21, also named CFIm25), an RNA‐binding protein, plays a critical role in regulating the expression of SASP factors through alternative polyadenylation (APA). APA allows adding poly(A) tail at different sites of 3′ UTR and generates transcript isoforms with different 3′ UTR lengths. We found that NUDT21 was downregulated in aging and fibrotic lungs, particularly at the fibrotic foci of IPF lungs known to have abundant senescent myofibroblasts and collagens. NUDT21 knockdown in normal lung fibroblasts promoted the 3′ UTR shortening of several STAT3 signaling components and enhanced STAT3 phosphorylation and the expression of several SASPs, including interleukins, collagens, and matrix metalloproteinases (MMPs). Moreover, NUDT21 downregulation may be associated with increased fibroblast senescence and abnormal mitochondrial function. Importantly, mice with *Nudt21* deletion in Col1a1 expressing cells aggravated bleomycin‐induced pulmonary fibrosis. Taking together, our study demonstrated an important role of NUDT21‐mediated APA in regulating SASP expression and fibroblast senescence that could contribute to the pathogenesis of IPF.

## Graphical abstract

In aging or fibrotic fibroblasts, NUDT21 is downregulated, leading to alternative polyadenylation/3′ UTR shortening of STAT3 pathway genes, enhancing their protein expression and promoting STAT3 phosphorylation. Activated STAT3 signaling subsequently drives the expression and release of senescence‐associated secretory phenotype (SASP) factors, thereby amplifying cellular senescence and contributing to exacerbated lung fibrosis.

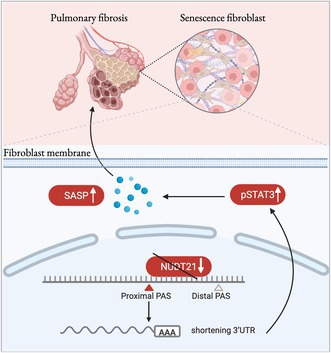

**Abbreviations**
3′ UTR: 3′ untranslated region
APA: alternative polyadenylation
BAL: Bronchoalveolar Lavage
BALF: Bronchoalveolar Lavage Fluid
CDS: coding region
CFIm: cleavage factor Im
COL1A1: collagen type I alpha 1
CPEB: cytoplasmic polyadenylation element binding protein
Crs: dynamic compliance
Cst: static compliance
DMEM: Dulbecco's Modified Eagle Medium
EF‐1a: elongation factor‐1 alpha
EGFR: epidermal growth factor receptor
EMEM: eagle's minimum essential medium
Ers: Elastance
FBS: fetal bovine serum
FN1: fibronectin 1
FOT: forced oscillatory pressure
FZD2: frizzled class receptor 2
G: tissue damping
H: tissue elastance
H₂O₂‐: hydrogen peroxide
IL: interleukin
IL6R: interleukin‐6 receptor
INFAR1: interferon alpha receptor 1
IPF: idiopathic pulmonary fibrosis
IRB: Institutional Review Board
IRES: internal ribosome entry site
JAK/STAT: janus kinase/signal transducer and activator of transcription
JAK2: janus kinase 2
LMNB1: Lamin B1
MMPs: matrix metalloproteinases
NUDT21: Nudix Hydrolase 21 (also named CFIm25)
OCR: oxygen consumption rate
P16: cyclin‐dependent kinase inhibitor 2A
P21: cyclin‐dependent kinase inhibitor 1A
P53: tumor protein P53
PAS: polyadenylation sites
PCR: polymerase chain reaction
PDL: population doubling level
PVDF: polyvinylidene fluoride
RIPA: radioimmunoprecipitation assay
Rn: Newtonian Airway Resistance
ROS: reactive oxygen species
Rrs: respiratory system resistance
RT: room temperature
SASP: senescence‐associated secretory phenotype
SA‐β‐gal: senescence‐associated beta‐galactosidase
SRSF3: serine and arginine rich splicing factor 3
STAT3: signal transducer and activator of transcription 3
TGFBR1: transforming growth factor beta receptor 1
U1 snRNA: U1 small nuclear RNA
WT: wild‐type
α‐SMA: alpha‐smooth muscle actin

## 1 Introduction

Idiopathic pulmonary fibrosis (IPF) is a lethal lung disease characterized by chronic, progressive, and irreversible scarring of the lungs. It is the most common form of interstitial lung disease, typically affecting older adults with an age of diagnosis ranging from 55 to 75 years old and a median age of 66 years old (Thannickal et al. 2004; Zank et al. 2018). Moreover, older patients tend to suffer a worse prognosis, higher mortality rate, and shorter survival time compared to younger patients (Pardo and Selman 2016). Particularly in the last two decades, there has been an increase in IPF prevalence and incidence rates among the elderly (Leung et al. 2015), suggesting a close link between aging and IPF. In the current rapidly aging society, there is an urgent need for effective pharmacologic therapies against IPF (Karimi‐Shah and Chowdhury 2015; Albert and Schwartz 2019). Furthermore, therapeutic options such as Pirfenidone and nintedanib have demonstrated effectiveness in numerous animal models of fibrosis; however, in patients with idiopathic pulmonary fibrosis (IPF), these drugs merely slow disease progression without providing a cure (Schaefer et al. 2011; Wollin et al. 2014). A key reason for this discrepancy may be that most preclinical studies are conducted in young mice, which do not fully replicate the complex features of IPF in human patients. Therefore, it is imperative to decipher aging‐dependent changes in IPF and identify novel mechanisms linking aging and IPF.

Aging is closely associated with cellular senescence, a state of permanent cell cycle arrest triggered by age‐related hallmarks such as oxidative stress, telomere shortening, and DNA damage (van Deursen 2014; Munoz‐Espin and Serrano 2014). Cellular senescence plays a key role in many aging‐related diseases, including IPF (Hecker et al. 2014; Shivshankar et al. 2012; Waters et al. 2018). It has been shown that aged mice revealed more persistent pulmonary fibrosis associated with an increased number of senescent fibroblasts compared to young mice (Hecker et al. 2014). Moreover, anti‐senescence agents ameliorated bleomycin‐induced lung fibrosis in mice (Schafer et al. 2017), suggesting that fibroblast senescence is a key risk factor that triggers pulmonary fibrosis pathogenesis. Senescent fibroblasts are metabolically active and exhibit a senescence‐associated secretory phenotype (SASP) that includes the secretion of inflammatory cytokines, chemokines, extracellular matrix remodeling factors, and growth factors (Tchkonia et al. 2013). These SASP factors have detrimental effects on tissue remodeling and contribute to the pathogenesis of IPF (Schafer et al. 2017). Despite these findings, the factors regulating the expression of SASP factors in senescent cells are unclear.

One emerging area of interest in this context is alternative polyadenylation (APA), a post‐transcriptional process that enables a single gene to produce transcripts with varying 3′ untranslated region (UTR) lengths, or, in rare cases, entirely different transcripts by attaching poly(A) tails at alternative polyadenylation sites (PAS) (Elkon et al. 2013; Di Giammartino et al. 2011). More than 50% of mammalian genes contain more than one PAS and can be regulated by APA (Elkon et al. 2013; Tian and Manley 2017). APA has been reported to play regulatory roles in various human physiological conditions and diseases including development (Ji et al. 2009), cancers (Xiang et al. 2017; Xia et al. 2014; Lembo et al. 2012), cardiac disorders (Creemers et al. 2016), and immune responses (Takagaki et al. 1996; Chuvpilo et al. 1999; Shell et al. 2005). APA also contributes to numerous cellular processes including cell proliferation (Sandberg et al. 2008; Staff 2016; Elkon et al. 2012), cell fate determination (Ji and Tian 2009; Brumbaugh et al. 2018), and cell senescence (Han et al. 2015). The selection of poly(A) sites can be determined not only by adjacent sequence elements but also by the levels of polyadenylation protein complexes. NUDT21 (also named CFIm25), a key component of the Cleavage Factor Im complex (CFIm), recognizes UGUA motifs upstream of the cleavage site. NUDT21 was identified by our group and others as a master APA regulator whose downregulation induces the most dramatic 3′ UTR shortening of mRNAs (Masamha et al. 2014; Kubo et al. 2006; Weng et al. 2020; Tseng et al. 2022). In general, shorter variants escape miRNA/3′ UTR binding protein‐mediated suppression and are normally more stable, leading to increased protein translation. Previous studies from our group demonstrated that NUDT21 deletion in fibroblasts promotes bleomycin‐induced skin and lung fibrosis in young mice (8–10 weeks, approximately equivalent to 20 years in human age) (Weng et al. 2019). However, whether NUDT21 is dysregulated in aging is not known. The main goal of this study is to investigate the role of NUDT21‐mediated APA as a novel mechanism linking aging to IPF.

## 2 Material and Methods

### 2.1 Human Samples

Explanted and deidentified lung tissue from discarded donors (controls) and patients with IPF was obtained from the UTHealth Houston Pulmonary Center of Excellence. The protocol for human sample collection was approved by institutional review boards (IRBs) (HSC‐MS‐15–1049 and HSC‐MS‐08‐0354) which adhere to the Declaration of Helsinki. IPF lung tissue was collected during lung explantation surgery and processed on‐site within 60 min. Discarded donor lungs (controls) devoid of acute or chronic pulmonary disease were obtained from Life Gift (Houston, TX). Detailed demographic and clinical data for both control and IPF‐derived tissues can be found in Table S1 within the supplementary data.

### 2.2 Mice Generation and Treatment

All mice in this study had a C57BL/6J genetic background. Mice were maintained under pathogen‐free conditions at the University of Texas Health McGovern Medical School in Houston, TX, with all experimental procedures receiving prior approval from the University of Texas Health Animal Welfare Committee (AWC 20‐0014). Wild‐type (WT) and Col1a1‐CreERT2 transgenic mice (B6.Cg‐Tg(Col1a1‐cre/ERT2)1Crm/J) were purchased from the Jackson Laboratory. NUDT21 floxed allele mice (Nudt21^f/f^, also named CFIm25^f/f^) were gifted from Dr. Michael Blackburn at the University of Texas McGovern Medical School in Houston. It was generated by deleting exons 2 and 3 of the NUDT21 gene (Weng et al. 2020; Weng et al. 2019) (Ozgene). To selectively knock down NUDT21 in fibroblasts, 8‐week‐old transgenic mice (Nudt21^f/f^Col1a1‐Cre) or age‐ and gender‐matched littermate controls (Col1a1‐creERT2) were administered intraperitoneal (i.p.) tamoxifen at 75 mg/kg/day for 3 consecutive days each month until 18 months for collection. Seven days after the last tamoxifen treatment, lung fibrosis was induced through repeated i.p. bleomycin injections (0.02 U/g/day) administered twice weekly for 4 weeks. For experiments in WT mice, lung fibrosis was induced through repeated i.p. bleomycin injections (0.035 U/g/day) administered twice weekly for 4 weeks.

### 2.3 Fibroblast Isolation and Culture

Human lung fibroblast cell line CCD8Lu was procured from ATCC (Manassas, VA). CCD8Lu cells were cultured in Eagle's Minimum Essential Medium (EMEM) supplemented with 10% fetal bovine serum (FBS) and 1% antibiotics. To knock down NUDT21 or JAK2, cells were cultured in antibiotic‐free media and transfected with 50 pmole/mL of control, or NUDT21 or JAK2 siRNA (Sigma) using Lipofectamine RNAiMAX (Life Technology, Grand Island, NY) on Day 0 and 1 (Weng et al. 2019; Ko et al. 2019). Cells were collected on Day 3 for western blot or real‐time PCR analysis. To inhibit the STAT3 signaling, fibroblasts were transfected with control or NUDT21 siRNA as described above. On Day 4, cells were treated with 20 μM STATTIC (R&D Systems) for 6 h and then collected for PCR analysis.

Primary human lung fibroblasts were isolated directly from the upper lobe of explanted IPF or control lungs using the explant technique (Hanmandlu et al. 2022). Similarly, primary mouse lung fibroblasts were isolated from the lungs of young (2 ~ 3 months old) and old mice (~18 months old) or mice treated with repetitive bleomycin using the explant method. Briefly, fresh lung tissue was cut into small fragments (~1 mm^3^) and plated onto culture dishes with a minimal amount of fibroblast growth medium (Dulbecco's Modified Eagle Medium (DMEM) supplemented with 10% fetal bovine serum (FBS) and 1% antibiotics) to prevent tissue floating. The tissue fragments were incubated at 37°C with 5% CO_2_, allowing fibroblasts to migrate out and adhere to the dish over 3–7 days. Once fibroblasts proliferated and reached ~80% confluence, the tissue pieces were removed, and cells were passaged for further expansion. Human fibroblasts at passages 5 and 6 and mouse fibroblasts at passages 1–3 were used for experiments.

### 2.4 Fibroblast Staining

Human primary lung fibroblasts were fixed in 4% paraformaldehyde and permeabilized with PBS + 0.05% Triton. The cells were blocked for 1 h in PBS with 3% Bovine Serum Albumin (catalog # BP1600) and then incubated in primary antibodies overnight at 4°C. The next day, the cells were washed twice for 5 min, followed by PBS/BSA for 5 min, and then incubated with the secondary antibodies for another hour at room temperature. After incubation, the cells were washed with PBS three times for 5 min. Finally, the sections were mounted with Aquamount (18606, Polysciences) and stored at 4°C. The following antibodies were used for immunostaining: rat anti‐VIMENTIN (1:50; R&D, MAB2105), rabbit anti‐PDGFRB (1:100; Cell Signaling 3169S), rabbit anti‐gamma H2A.X (1:200, Cell Signaling 9718S), PE anti‐human CD90 antibody (Biolegend, 5E10). Secondary antibodies from Jackson ImmunoResearch were used: Alexa 488‐, Cy3‐conjugated donkey anti‐rabbit (1:1000; 711‐545‐152,711‐165‐152), Alexa 488‐conjugated donkey anti‐rat (1:1000, 712‐545‐153). Images were taken with an Olympus FV1000 confocal microscope.

### 2.5 Reactive Oxygen Species (ROS) Assay

Intracellular ROS levels were measured using the DCFDA/H2DCFDA—Cellular ROS Assay Kit (Abcam, ab113851) following the manufacturer's protocol with minor modifications. Briefly, cells were seeded in 6‐well plates and transfected with control or NUDT21 siRNA as described above. On day 4 after transfection, cells were detached using trypsin–EDTA and incubated with 10 μM 2′,7′–dichlorofluorescin diacetate (DCFDA) in serum‐free medium at 37°C for 30 min in the dark. After incubation, cells were washed to remove excess dye and analyzed using a BD LSRFortessa cytometer. Background fluorescence was determined using unstained or DCFDA‐negative control samples. The percentage of high DCFDA expression cells was determined.

### 2.6 Lung Function Assay

The mouse lung function was analyzed using the flexiVent FX system, equipped with the FX1 module and Flexiware v8.3 software. Mice were anesthetized with an i.p. injection of 5% Avertin (0.012 mL/g body weight). Once a surgical plane of anesthesia was reached, a tracheostomy was performed, and the trachea was cannulated with an 18‐gauge cannula. Then the lung was inflated to 30 cmH_2_O over 6 s and returned to normal ventilation for 1 min. Respiratory mechanics were measured under closed chest conditions utilizing forced oscillatory pressure (FOT) and broadband oscillation techniques. Parameters assessed included respiratory system resistance (Rrs), dynamic compliance (Crs), elastance (Ers), and Newtonian airway resistance (Rn) generated via a ramp‐style pressure‐driven maneuver to a maximum of 30 cmH_2_O to calculate static compliance (Cst). Three independent measurements were carried out for each mouse, and the mean values of these measurements were calculated for the final parameter comparison.

### 2.7 Inflammation and Cellular Differential Assay

Mouse lungs were lavaged with 0.4 mL sterile PBS three times to collect bronchoalveolar lavage fluid (BAL). Total cell numbers in BAL were determined using a hemocytometer. For the differential assay, BAL cells were spun onto microscope slides at 1200 rpm for 5 min, and stained with Diff‐Quick (Dade Behring, Deerfield, IL) to identify immune cell types in the BAL. The percentages of macrophages, lymphocytes, and neutrophils were assessed in a blind manner for the stained slides, and the number of each cell type was calculated by multiplying their percentage by the total BAL cell numbers.

### 2.8 Western Blot

Collected lung samples were pulverized using a Tissue Pulverizer (BioSpec Products) in liquid nitrogen. Cell or pulverized lung tissue samples were lysed in RIPA buffer (Boston Bioproducts), separated using 4%–20% Mini‐PROTEAN TGX Precast Protein Gels (Bio‐RAD), and transferred to a PVDF membrane (Millipore Sigma). After blocking with EveryBlot Blocking Buffer (BioRad) for 10 min at room temperature, membranes were incubated with primary rabbit anti‐NUDT21, rabbit anti‐CPSF6, rabbit anti‐CPFS7 (Proteintech), rabbit anti‐Collagen I (Abcam), rabbit anti‐Fibronectin (Sigma Aldrich), mouse anti‐β‐Actin antibodies (Sigma‐Aldrich), or mouse anti‐α‐GAPDH (Life Technology) antibodies overnight at 4°C. After rinsing, membranes were rocked in corresponding secondary horseradish peroxidase‐conjugated antibodies for 1 h at room temperature (Cell Signaling Technology). The luminescence signal was detected in Amersham ECL Prime Western Blotting Detection Reagent (GE Healthcare Bio‐Sciences), and images were captured using a Bio‐Rad Chemidoc imager.

### 2.9 Real‐Time Quantitative PCR

Total RNA was extracted using the Qiagen RNAeasy kit. A step during the extraction was included to remove the genomic DNA. Purified RNA was then reverse transcribed using Reverse Transcription Supermix (Bio‐Rad). Real‐time PCR was performed using a BioRad CFX384 PCR Detection System and primers listed in Table S2. Data were quantified using the relative Ct method and presented as the mean ratio to β‐actin or 18sRNA.

### 2.10 Immunohistochemistry

Formaldehyde‐fixed and paraffin‐embedded human or mouse lungs were sectioned (4 μm). Lung sections were deparaffinized in Histo‐Clear (Pational Diagnostics), followed by rehydration through a series of graded alcohols (100%, 95%, 80%, 70%, 50%, and 0%). Rehydrated slides were incubated in 1X Tris‐Based antigen unmasking solution (VectorLabs, Burlingame, CA) at 95°C for 20 min, and blocked with BLOXALL Endogenous Blocking Solution (VectorLabs) for 10 min followed by 5% normal goat serum for 1 h at room temperature. Then slides were rocked in primary rabbit anti‐NUDT21 antibodies (1:200) overnight at 4°C, followed by ImmPRESS HRP Horse Anti‐Rabbit antibodies (VectorLabs) for 1 h at room temperature (RT). After rinsing, slides were developed with ImmPACT DAB EqV Substrate Kit (VectorLabs). For counterstaining with a‐SMA, the stained slides were blocked in 5% normal goat serum for 1 h at room temperature, followed by overnight incubation with primary anti‐αSMA antibodies at 4°C and 1 h incubation with ImmPRESS Alkaline phosphatase Horse Anti‐Rabbit antibodies (VectorLabs) for 1 h. Slides were then developed with Vector Red AP substrate, and co‐stained with hematoxylin (VectorLabs). Finally, slides were dehydrated through graded alcohol solutions (70%, 95%, 100%) and mounted using CytoSeal Mounting Medium (Electron Microscopy Sciences).

### 2.11 Masson's Trichrome and Ashcroft Assay

Masson's Trichrome staining was carried out using the Trichrome Stain (Masson) Kit from Sigma‐Aldrich. Briefly, slides were deparaffinized, rehydrated, and then stained with Weigert's iron hematoxylin for 5 min, followed by Scarlet‐Acid Fucshin for 5 min. After rinsing, the slides were incubated in working Phosphotungstic/Phosphomolybdic Acid Solution for 5 min and in aniline blue for 15 min, and then rinsed in 1% acetic acid. Finally, slides were dehydrated and mounted in CytoSeal Mounting Medium. The assessment of fibrosis severity was executed using Ashcroft scores, which were evaluated independently by two individuals, blinded to the experimental conditions, utilizing Masson's Trichrome stained slides. A modified grading system (with scores 8 and 9) was applied, and a minimum of 10 randomly selected areas within each lung specimen were assessed for fibrotic changes.

### 2.12 Statistics

For data following a normal distribution, a two‐tailed Student *t*‐test was used to compare two treatment groups, and ANOVA was used to determine significant differences among more than two treatment groups followed by multiple comparisons to determine significant differences between any two treatments. For data that did not follow a normal distribution, nonparametric analysis methods were employed to calculate *p*‐values. The significance level was set at *p* < 0.05, and results were presented as Mean ± Mean Squared Error (MSE).

## 3 Results

### 3.1 NUDT21 Is Downregulated in Human Fibrotic and Aging Lungs

**FIGURE 1:**
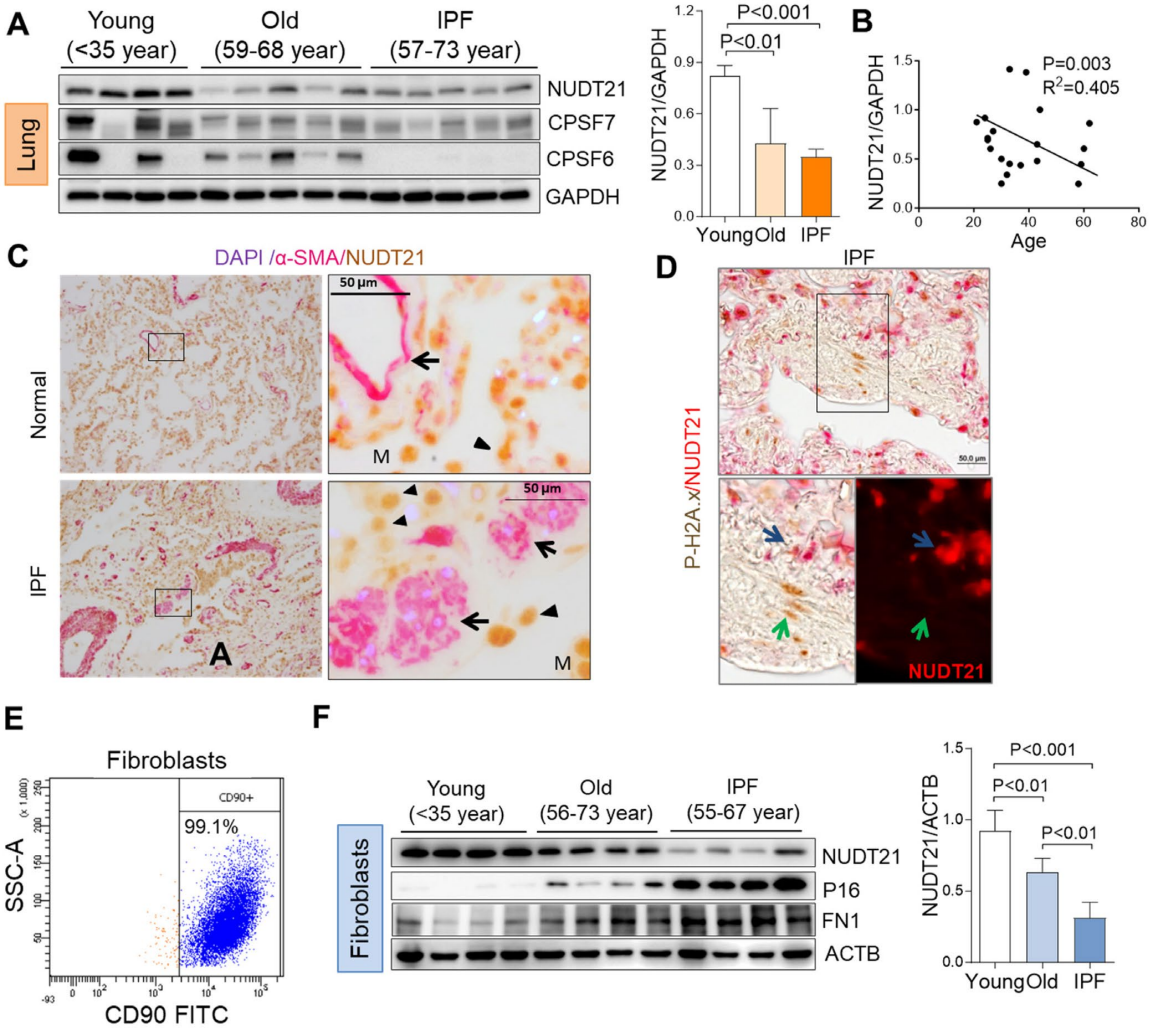
NUDT21 expression is decreased in IPF and aging human lungs and fibroblasts. (A) Western blot (left panel) and densitometry analysis (right panel) show NUDT21 protein levels in the lungs of young donors (< 35‐year‐old), aging donors (59–68 year‐old), and IPF patients (57–73 year‐old). (B) Linear regression of western blot densitometry shows NUDT21 protein levels in 20 normal human lungs are negatively correlated with age. (C) Dual‐immunostainings showing NUDT21 (arrowhead) are mainly downregulated in α‐SMA positive fibroblasts (arrow) in IPF lungs. M, macrophages. (D) Dual‐immunostainings show phospho‐H2A.x (brown) staining in fibrotic foci (green arrow) where NUDT21 (Red) expression is missing in IPF lungs. (E) Flow cytometry of CD90 labeled isolated primary fibroblasts showing 99% purity. (F) Western blot (left panel) and densitometry analysis (right panel) showing NUDT21 levels in lung fibroblasts isolated from young and old donors, as well as IPF patients. All of the densitometry data were presented as the ratio of NUDT21 to GAPDH or ATCB. The *p*‐values were calculated from a one‐way ANOVA followed by Dunnett's multiple comparisons test.

To understand whether APA plays a role in IPF development, we first examined the protein expression of the key APA regulator NUDT21 in IPF and control lungs. Decreased NUDT21 protein levels were detected in IPF lungs and age‐matched donor lungs compared to the lungs of young donors (Figure 1A). The levels of Cleavage and Polyadenylation Specific Factor 6 (CPSF6) and CPSF7—cofactors of NUDT21 within the cleavage factor Im (CFIm) complex—also showed a trend toward downregulation in aged and IPF lungs, though these changes were less consistent than those observed for NUDT21 (Figure 1A). In contrast, the expression of other APA‐related components, including CPSF3 (also known as CPSF73), FIP1 like 1 (FIP1L1), symplekin (SYMPK), and polyadenylate‐binding nuclear protein 1 (PABPN1), did not change significantly in both aged and IPF lungs (Figure S1A). Therefore, we focused on NUDT21 for further studies. Western blot of 20 normal lungs at different ages suggests a negative correlation between NUDT21 protein levels and age (Figure 1B). Dual immunostaining indicated that NUDT21 downregulation was mainly observed in alpha‐smooth muscle actin (α‐SMA) positive myofibroblasts in IPF lungs (Weng et al. 2019) (Figure 1C). Fibroblast senescence is a hallmark of aging, and it is detrimental to IPF pathogenesis (Schafer et al. 2017). It has been shown that IPF fibroblasts have a high expression of the senescent marker P16 (Schafer et al. 2017) and exhibit a senescent phenotype (Alvarez et al. 2017). Similarly, we observed positive staining of a senescent marker, phospho‐Histone H2A.X (Ser139) (P‐H2A.x), in fibrotic foci of IPF lungs where NUDT21 expression was low (Figure 1D green arrow). Furthermore, we isolated primary lung fibroblasts from young and old control lungs and IPF lungs. Flow cytometry with CD90 antibodies demonstrated > 95% purity of isolated fibroblasts (Figure 1E). Consistently, immunofluorescence staining for vimentin and platelet‐derived growth factor receptor beta (PDGFRβ) demonstrated that nearly all cells were positive for both markers, further confirming the high purity of the isolated fibroblasts (Figure S1B). The levels of senescent marker P16 and fibrotic marker FN1 were elevated in aging fibroblasts and further enhanced in IPF fibroblasts (Figure 1F). Interestingly, NUDT21 protein was downregulated in fibroblasts from old donors, and it was further decreased in IPF fibroblasts (Figure 1F). Similarly, CPSF6, the cofactor of NUDT21, was diminished in aged fibroblasts and further decreased in those from IPF lungs (Figure S1C). In contrast, the expression of other APA‐related factors, including CPSF7, FIP1L1, and SYMPK, remained unchanged (Figure S1C). Taken together, our data demonstrate that NUDT21 is consistently downregulated in fibroblasts from aged and IPF lungs, supporting a mechanistic link between aging and IPF pathogenesis. Myofibroblasts are a key player in pulmonary fibrosis, responsible for excessive extracellular matrix protein (ECM) deposition (Thannickal et al. 2004; Pardo and Selman 2016). Our results suggest that NUDT21 downregulation in this cell population could be a crucial driver of fibrosis. Therefore, we focused on examining NUDT21 signaling in fibroblasts for the following studies.

### 3.2 NUDT21 Is Downregulated in Murine Fibrotic and Aging Lungs

**FIGURE 2:**
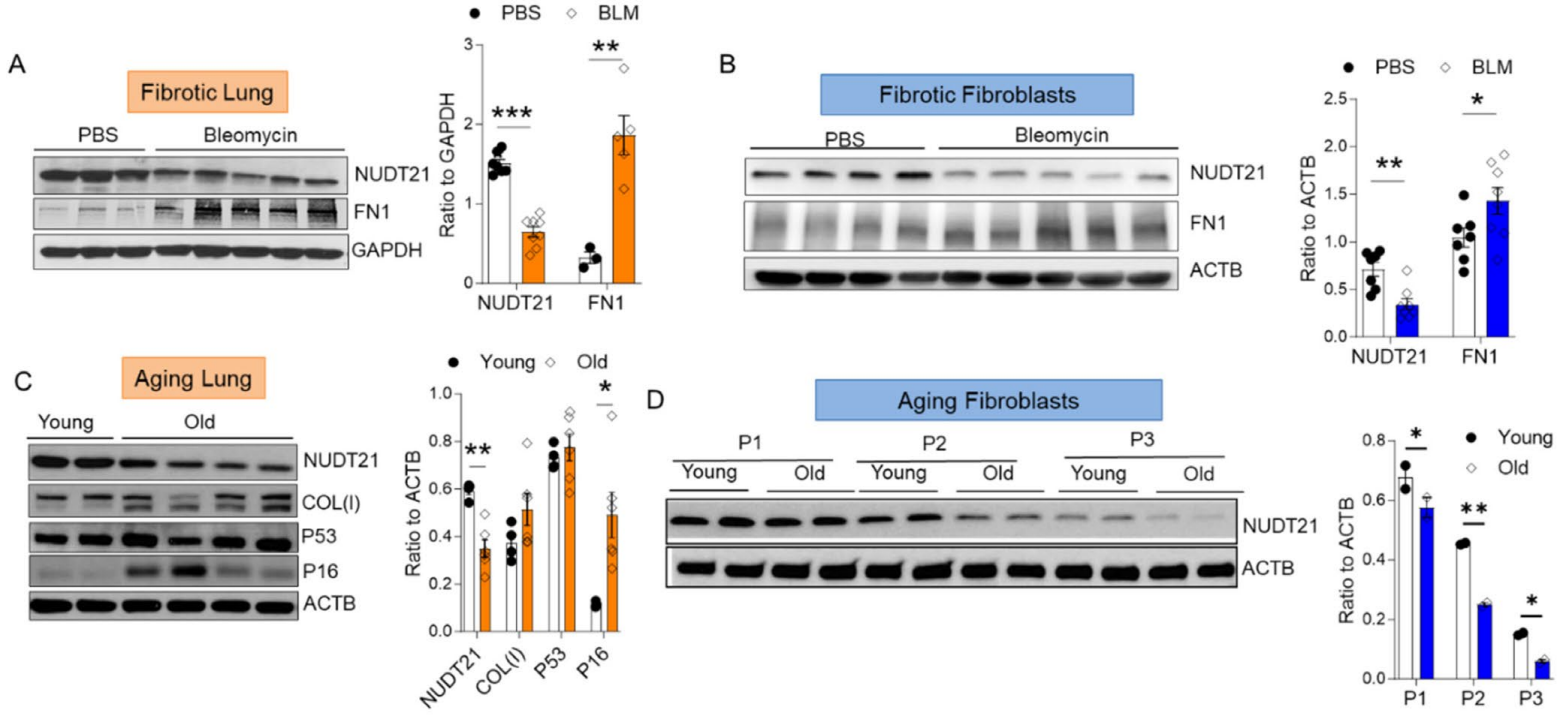
NUDT21 is decreased in mouse fibrotic and aging lungs. Wildtype (WT) C57BL6 mice were injected with 0.035 μ/g bleomycin bi‐weekly to induce pulmonary fibrosis, and the lungs were analyzed on Day 33. NUDT21 protein expression was determined using Western blot in the lung (A) or primary lung fibroblasts (C) from mice injected with PBS or bleomycin; and lung (B) or primary lung fibroblasts (D) from young (2‐month‐old) and old (18‐month‐old) WT mice. Fibronectin (FN1) or Collagen (COLI) were used as fibrotic markers, and P53 and P16 were used as senescent markers. GAPDH or ACTB were used as internal controls.

To investigate the role of NUDT21 in murine models of pulmonary fibrosis, we used a well‐developed repetitive intraperitoneal (i.p.) bleomycin pulmonary fibrosis model to induce pulmonary fibrosis in mice (Zhou et al. 2011; Luo et al. 2016; Moore and Hogaboam 2008). Prominent pulmonary fibrosis was observed on Day 33 after the first bleomycin injection as shown by increased fibronectin (FN) levels, and NUDT21 protein levels were reduced in bleomycin‐treated lungs (Figure 2A). Similar to our observations in human lungs, NUDT21 level was reduced in primary fibroblasts isolated from the lungs of bleomycin‐treated mice (Figure 2B). Additionally, NUDT21 levels were decreased in the lungs of old mice (18 months) in association with increased expression of collagen I COL(I) and senescent markers P53 and P16 (Figure 2C). To determine whether NUDT21 levels are dysregulated in aging lungs, we isolated primary lung fibroblasts from young (2 months old) and old (18 months old) mice and cultured them for three passages. While only a slight difference in NUDT21 levels was observed between young and old fibroblasts at passage 1, its levels decreased over time in cultured young fibroblasts, and declined more rapidly in old fibroblasts compared to young ones (Figure 2D). These findings suggest that NUDT21 levels decrease during fibroblast senescence and that fibroblasts from aged mouse lungs undergo senescence more rapidly than those from younger mice. Overall, these data suggest that NUDT21 is downregulated in fibroblasts from aging and fibrotic mouse lungs.

### 3.3 NUDT21 Is Downregulated in Senescent Fibroblasts In Vitro

**FIGURE 3:**
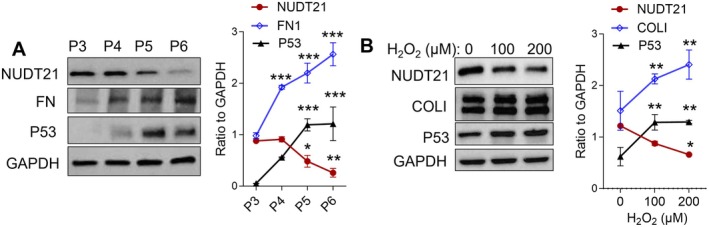
NUDT21 is downregulated in senescent fibroblasts. (A) Normal human lung fibroblasts (started at passage 3) were subcultured at a 1:4 ratio every 2–4 days as they reached ~80%–90% confluence. Cells were collected at each passage until they ceased replication at passage 6. Western blot was performed to analyze the protein levels of NUDT21, FN, P53, and GAPDH. (B) Normal human lung fibroblasts were exposed to either PBS or 100–200 μM H_2_O_2_ for 2 h, then collected for analysis 7–10 days later. Right panel: Representative western blot image showing NUDT21, P53, and COL(I) expression. Left panel: Densitometry analysis of western blot images. *p < 0.05, **p < 0.01 and ***p < 0.001 versus P3 or 0 μM H_2_O_2_ calculated from a two‐tailed student *t*‐test.

To assess whether NUDT21 is dysregulated in senescent fibroblasts, we used two established models in vitro: the replicative senescence model (Dimri and Campisi 1994), driven by telomere shortening, and the H_2_O_2_‐induced senescence model through oxidative stress (Chen and Ames 1994). In the replicative senescence model, fibroblasts (initiated at passage 3) were subcultured at a 1:4 ratio every 2–4 days as they reached ~80%–90% confluence. In our study, cells were considered senescent at passage 6, when population doubling levels (PDL, typically > 60 at senescence) plateaued across successive subcultures. For the H_2_O_2_‐induced model, fibroblasts were exposed to either PBS or 100–200 μM H_2_O_2_ for 2 h, then collected for analysis 7–10 days later. We observed that NUDT21 expression gradually declined in cultured normal primary lung fibroblasts (CCD8Lu) as they ceased replication (Figure 3A), and this process is associated with increased levels of FN1 and P53 (Figure 3A). Similarly, H_2_O_2_‐treated cells showed suppressed NUDT21 levels, along with increased COL(I) and P53 expression (Figure 3B). Collectively, these results indicate that NUDT21 expression decreases in senescent fibroblasts.

### 3.4 NUDT21 Downregulation Is Associated With Increased STAT3 Activation and SASP Expression

**FIGURE 4:**
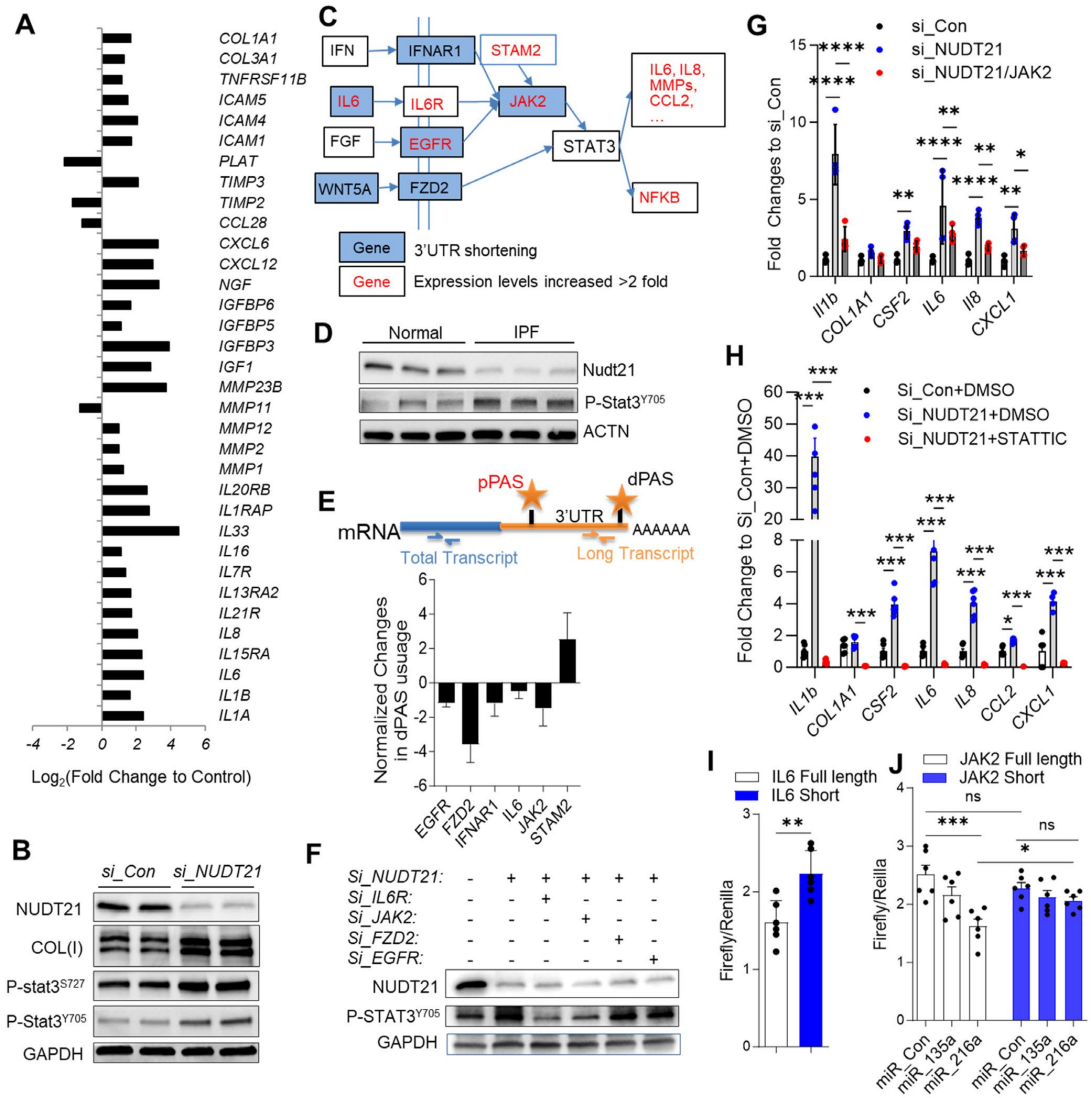
NUDT21 Knockdown promotes STAT3 activation. Normal human lung fibroblasts were transfected with control or NUDT21 siRNA. After 3 days, fibroblasts were collected for RT qPCR, protein or RNA‐sequencing (GSE137276) (Weng et al. 2019). (A) RNA‐seq revealed increased expression of several SASP factors. (B) Western blots were performed to determine the protein levels of NUDT21, COLI, phosphorylated STAT3 (P‐stat3^S727^ and P‐STAT3^Y705^), as well as GAPDH in control or NUDT21 knockdown fibroblasts. (C) Pathway analysis showing key upstream components of the STAT3 pathway having 3′ UTR shortening (highlighted with blue) and/or increased transcript expression (red) in *NUDT21* knockdown fibroblasts, and downstream genes having increased expression (red). (D) Western blots were performed to determine the protein levels of NUDT21, P‐STAT3^Y705^ and GAPDH in primary fibroblasts isolated from normal donors or IPF patients. (E) The upper panel demonstrates primer locations for monitoring APA. The lower panel demonstrates the 3′ UTR shortening of genes involved in the STAT3 pathway. (F) Western blot showing an increased STAT3 phosphorylation by *NUDT21* siRNA that can be suppressed by knocking down genes (IL6R and JAK2) involved in STAT3 pathway. (G) RT qPCR showed SASP factor expression in cells transfected with control siRNA, NUDT21 siRNA, or both NUDT21 and JAK2 siRNA. Samples were analyzed 4 days after transfection. (H) Fibroblasts were transfected with control or NUDT21 siRNA. Four days later, cells were treated with STAT3 inhibitor STATTICS (10μM) for 6 h and collected for RT qPCR analysis of SASPs levels. (I) Fibroblasts were transfected with psiCHECK‐2 plasmids containing either the full‐length or short IL6 3′ UTR cloned downstream of the firefly luciferase gene. After 24 h, luciferase activity was measured. (J) Fibroblasts were co‐transfected with control, miR‐135a, or miR‐216a mimics along with psiCHECK‐2 plasmids containing the long or short JAK2 3′ UTR. Luciferase activity was measured 24 h post‐transfection. *p < 0.05, **p < 0.01 and ***p < 0.001 and ****p < 0.001.

To understand the functional consequence of NUDT21 downregulation on fibroblasts, we next used RNA‐Seq for transcriptomic and APA profiling of NUDT21 knockdown lung fibroblasts (Weng et al. 2019). Fibroblasts with successful NUDT21 silencing showed increased expression of COL(I) (Figure 4A). Consistent with previous reports where NUDT21 depletion induced 3′ UTR shortening of target genes, we identified 808 genes with shortened 3′ UTR and only 29 genes with lengthened 3′ UTR (GSE108352) (Weng et al. 2019). RNA‐Seq also identified multiple SASP factors with increased transcript expression upon NUDT21 depletion, such as interleukins including IL6 and IL8, MMPs, chemokines, and collagens including COL1A1 (Figure 4B). Notably, among all elevated SASPs, only some, such as *COL1A1* and *IL6*, have 3′ UTR shortening (Weng et al. 2019), suggesting that other SASP factors are regulated by indirect pathways. KEGG pathway analysis of the APA genes and differentially expressed genes in *NUDT21* knockdown fibroblasts identified multiple genes involved in the STAT3 pathway having either 3′ UTR shortening (Figure 4C, highlighted in blue) or increased transcript levels (Figure 4C, red fonts). Consistently, phosphorylated STAT3 (P‐STAT3^Y705^) was elevated in NUDT21 knockdown fibroblasts (Figure 4A), suggesting that NUDT21 depletion induces signaling cascades to activate the STAT3 pathway. Notably, phosphorylated STAT3 (P‐STAT3^Y705^) was also elevated in primary IPF fibroblasts that have reduced NUDT21 expression (Figure 4D), suggesting a similar feature between IPF fibroblasts and normal fibroblasts with NUDT21 knockdown. We also adopted a PCR‐based method to monitor the percentage of distal polyadenylation site (dPAS) usage (Masamha et al. 2014; Weng et al. 2019). Briefly, two primer pairs were designed, with one targeting the very end of the 3′ UTR to represent the transcript with long 3′ UTR, and the other targeting the coding area to represent the total levels of the transcript (Figure 4D upper panel). The percentage of the long transcript was calculated as ΔCT = CT_distal_‐CT_total_. Final data are shown as Log_2_ (the percentage of long transcript in NUDT21 knockdown fibroblasts/the percentage of long transcript in control fibroblasts) (ΔΔCT = ΔCT_average of target_ − ΔCT_average of control_). A negative ΔΔCT means there is 3′ UTR shortening. As shown in Figure 4E, the 3′ UTR shortening of *EGFR, FZD2, IFNAR1, IL6, and JAK2* was confirmed in NUDT21 knockdown cells. Furthermore, the knockdown of STAT3 pathway genes, including IL6R or JAK2, together with NUDT21, can mitigate STAT3 activation induced upon NUDT21 depletion (Figure 4F). Consistently, silencing JAK2, a key component of the STAT3 pathway, suppressed the transcript levels of several SASPs (e.g., *Il6*, *Il1b*, *Il8*) induced by NUDT21 depletion (Figure 4G).

Moreover, to directly assess the role of STAT3 activation in NUDT21‐loss‐mediated SASP upregulation, we treated NUDT21‐depleted fibroblasts with the STAT3 inhibitor STATTIC. Notably, STATTIC treatment significantly reduced the expression of SASP genes induced by NUDT21 knockdown (Figure 4F). Finally, to investigate whether NUDT21‐loss–mediated 3′ UTR shortening directly affects protein expression, we cloned the long and short 3′ UTRs of IL6 and JAK2 downstream of the firefly luciferase gene in the psiCHECK‐2 vector, which also expresses Renilla luciferase for normalization of transfection efficiency. The construct containing the short IL6 3′ UTR significantly increased firefly luciferase activity compared to the long 3′ UTR, indicating enhanced translation (Figure 4I). However, no difference in luciferase activity was observed between the short and long JAK2 3′ UTR constructs (Figure 4J), possibly due to low endogenous expression of relevant miRNAs in HEK293T cells. To address this, we co‐transfected the JAK2 constructs with either miR‐135a or miR‐216a, which have predicted binding sites on the long but not short 3′ UTR. MiR‐135a had no effect, while miR‐216a significantly suppressed luciferase activity only in the long 3′ UTR construct. These results suggest that 3′ UTR shortening may enhance JAK2 expression by evading miRNA‐mediated repression, supporting a functional consequence of NUDT21 loss (Figure 4J). Furthermore, cells transfected with the short JAK2 3′ UTR construct and miR‐216a exhibited significantly higher luciferase activity than those transfected with the long 3′ UTR construct and miR‐216a (Figure 4J), suggesting that NUDT21‐mediated 3′ UTR shortening can enhance translational efficiency by removing inhibitory miRNA‐binding sites. Overall, these findings suggest that STAT3 activation in NUDT21 knockdown fibroblasts is important to induce SASP expression.

### 3.5 NUDT21 Depletion Promotes Fibroblast Senescence and Inhibits Mitochondrial Oxygen Consumption

**FIGURE 5:**
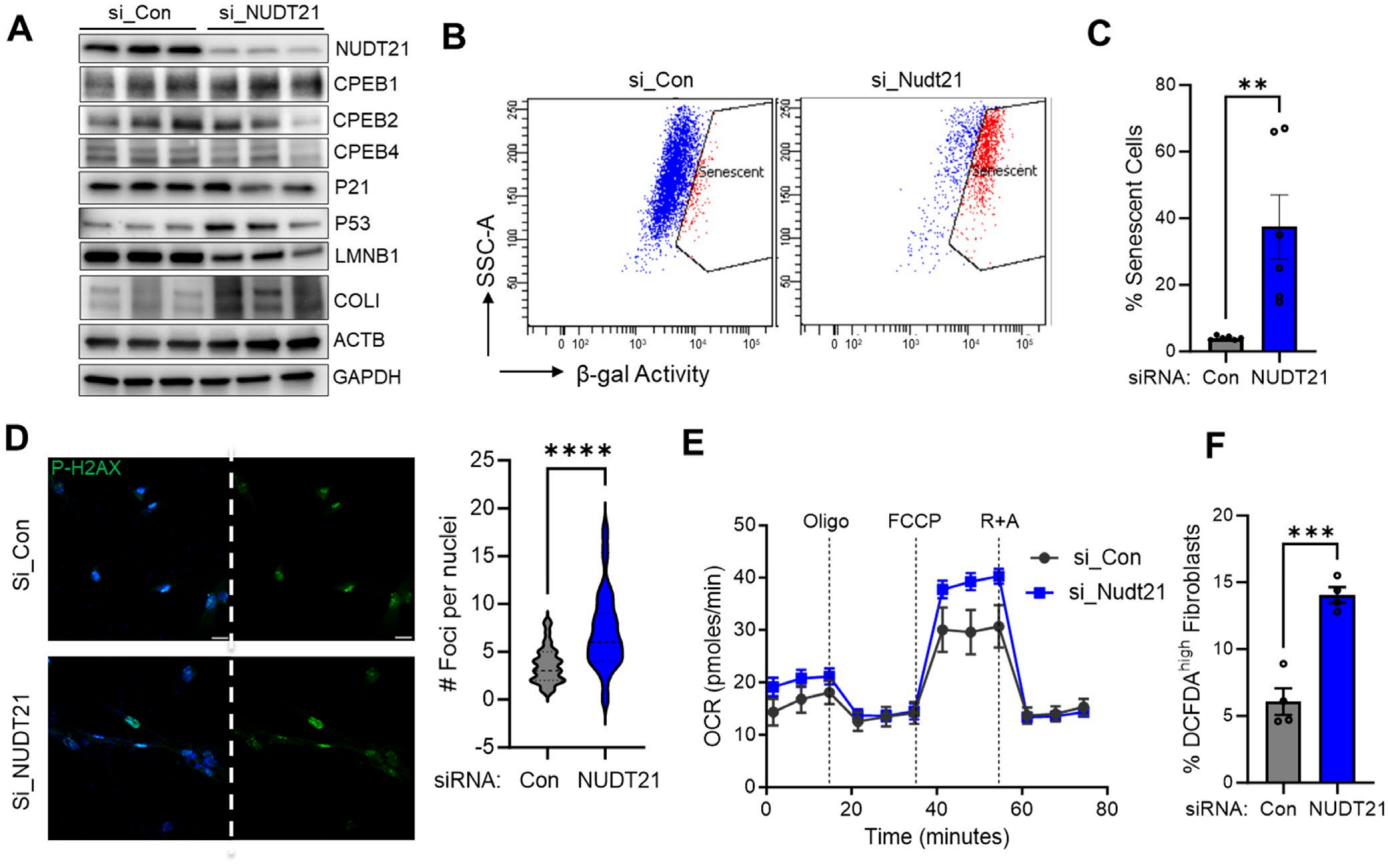
NUDT21 knockdown increases cell senescence. Normal human lung fibroblasts were transfected with control or NUDT21 siRNA and analyzed 5 days after transfection. (A) Western blots were performed to determine the protein levels of NUDT21, CPEB1, CPEB2, CPEB3, P21, P53, LMNB1, COLI, ACTB, and GAPDH in control or NUDT21 knockdown fibroblasts. (B) Flow cytometry was performed to detect the senescent cells using the CellEvent Senescence Green Detection Kit. The representative Flow cytometry image was shown. (C) Quantification of the percentage of senescent cells from the flow cytometry in B. (D) Fibroblasts were stained with anti‐p‐H2a.X antibodies to determine cell senescence. Left, representative images of p‐H2a.X (green) stained fibroblasts. DAPI blue staining was used to show the nuclei. Right: Quantification of the number of Foci per nucleus. Scale bar = 20 μm. (E) The Agilent Seahorse XF Mito Stress Test kit was used with the Agilent Seahorse XFe16 Extracellular Flux Analyzer to analyze oxygen consumption rate in control or NUDT21 knockdown fibroblasts. (F) DCFDA/H2DCFDA—Cellular ROS Assay Kit was used to stain the ROS in cells transfected with control or NUDT21 siRNA. Flow cytometry was performed to detect the ROS signal, and the percentage of cells with high DCFDA staining was quantified and plotted. *p < 0.01, **p < 0.001, ****p < 0.0001 versus si_Con.

While STAT3 activation is widely recognized to enhance tumor cell survival, recent studies have also revealed its role in promoting cellular senescence (Kojima et al. 2012). Our observation that STAT3 is activated in NUDT21 knockdown fibroblasts prompted us to investigate whether this activation contributes to cellular senescence. We first examined the protein expression of several senescence‐associated transcription factors and markers. While levels of cytoplasmic polyadenylation element‐binding proteins CPEB1, CPEB2, and CPEB4 remained unchanged, we observed decreased expression of lamin B1 (LMNB1) and increased expression of collagen type I (COL1) and TP53 (p53), indicating enhanced senescence (Figure 5A). Furthermore, using the CellEvent Senescence Green Detection Kit, we measured the activity of β‐galactosidase, a hallmark biomarker of cellular senescence. Consistently, β‐galactosidase activity was significantly elevated in NUDT21‐silenced fibroblasts (Figure 5B,C). We also performed immunofluorescence staining for phosphorylated histone H2A.X (p‐H2A.X) and quantified nuclear foci as an indicator of DNA damage. NUDT21 KD fibroblasts showed a significant increase in the number of p‐H2A.X foci (Figure 5D). Since senescent cells often exhibit mitochondrial dysfunction and increased production of reactive oxygen species (ROS), we performed a Seahorse analysis to assess mitochondrial function. We found that the oxygen consumption rate (OCR) was significantly increased in NUDT21‐depleted cells (Figure 5E), which aligns with previous findings of elevated OCR in senescent cells (Korolchuk et al. 2017). Additionally, ROS staining with the DCFDA/H2DCFDA—Cellular ROS Assay Kit revealed a significantly higher proportion of DCFDA‐high fibroblasts in the NUDT21 KD group compared to controls (Figure S2, Figure 5F). Together, these results indicate that NUDT21 knockdown promotes human lung fibroblasts toward a senescent phenotype.

### 3.6 NUDT21 Overexpression Attenuates SASP Upregulation in Fibroblasts

**FIGURE 6:**
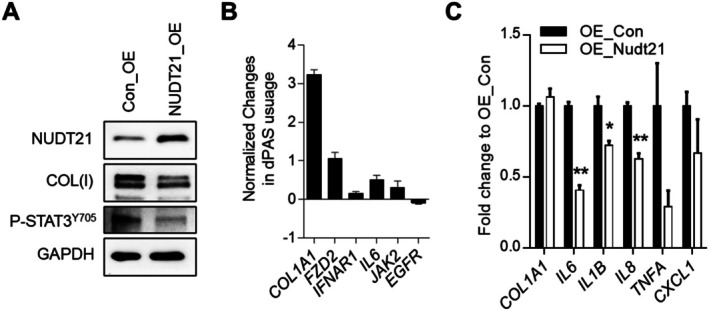
NUDT21 overexpression suppresses IPF fibroblast senescence and SASP expression. Control (express GPF) or NUDT21 overexpression (express NUDT21‐IRES‐GFP) lentiviruses were generated using the third generation Lentivirus Packing System and used to infect primary IPF fibroblasts at 25 MOI (multiplicity of infection). (A) Western blot showed the protein levels of NUDT21, COL(I), P‐STAT3^Y705^, and ACTB. (B) RT‐qPCR determined the distal polyA site usage of NUDT21 target genes. (C) RT‐qPCR demonstrated the transcript levels of SASPs in IPF lung fibroblasts with or without NUDT21 overexpression. *p < 0.05 and **p < 0.01 versus Con_OE based on a two‐tailed Student *t*‐test.

In a complementary gain‐of‐function approach, we investigated whether SASP expression can be alleviated by NUDT21 overexpression (OE) in fibroblasts. Specifically, we constructed a NUDT21 OE lentivirus vector by cloning the coding region (CDS) of human *NUDT21* into the pLV‐EF1a‐IRES‐Puro Vector that contains a human elongation factor‐1 alpha (EF‐1a) promoter upstream of an internal ribosome entry site (IRES) element to co‐express the puromycin marker. *NUDT21* CDS was inserted between the EF‐1a and IRES. The IRES allows the expression of *NUDT21* and the puromycin marker from a single mRNA, thus ensuring the co‐expression of NUDT21 and the puromycin marker in the same cells. The NUDT21 OE lentiviruses were then generated using the 3rd generation Lentivirus Packing System. NUDT21 OE lentiviruses successfully enhanced NUDT21 expression in IPF lung fibroblasts (Figure 6A). Increased NUDT21 was associated with reduced COL(I) and STAT3^Y705^ protein levels (Figure 6A), 3′ UTR shortening of several genes involved in the STAT3 pathway including *COL1A1*, *FZD2*, *INFAR1*, *IL6*, and *JAK2* (Figure 6B), and lower transcript levels of SASPs including *IL6*, *IL1B*, and *IL8* (Figure 6C). Notably, although the total level of *COL1A1* transcript did not decrease in NUDT21 OE cells (Figure 6C), it had 3′ UTR lengthening (Figure 6B) and decreased protein translation (Figure 6A), indicating that 3′ UTR lengthening of *COL1A1* is sufficient to downregulate its protein production without suppressing its total transcript level. Together, these data indicate that NUDT21 overexpression suppresses SASP gene expression and alleviates the senescent phenotype.

### 3.7 Fibroblast NUDT21 Deletion Aggravates Bleomycin‐Induced Pulmonary Fibrosis During Aging

Previous studies from our group have demonstrated that *Nudt21* deletion in *Col1a1* expressing cells, including lung fibroblasts (Rossert et al. 1995; Goodwin et al. 2018) (*Col1a1* ^*creERT2*^ *‐Nudt21* ^*f*/f^), exacerbated bleomycin‐induced lung fibrosis in young mice (Weng et al. 2019). To determine whether NUDT21‐loss mediated posttranscriptional changes in fibroblasts can lead to spontaneous fibrosis in aging, we continuously induced NUDT21 deletion by injecting 2‐month‐old *Col1a1* ^*creERT2*^ *‐Nudt21* ^*f*/f^ or control *Col1a1* ^*creERT2*^ mice with ~75 mg tamoxifen/kg body weight 5 consecutive days each month until 18 months for analysis. NUDT21 levels were decreased in *Col1a1* ^*creERT2*^ *‐Nudt21* ^*f*/f^ mice compared to control (Figure S3A). Since fibrotic responses often involve shared molecular mechanisms across multiple organs, we examined additional organs prone to fibrosis in systemic fibrotic conditions—skin, kidney, and liver—in addition to the lungs, to comprehensively assess NUDT21's role in fibroblast‐related fibrosis. Interestingly, reduction in NUDT21 did not lead to detectable increases in COL1 or FN1 in the lungs, skin, kidneys, or liver (Figure S3B). Moreover, histological and Masson's trichrome staining showed no significant differences in collagen accumulation or fibrosis in any of the organs studied, including the lungs, between groups (Figure S3C). These findings suggest that NUDT21 depletion alone does not induce spontaneous fibrosis during aging, and additional triggers may be required to drive fibrosis in the context of NUDT21 loss.

**FIGURE 7:**
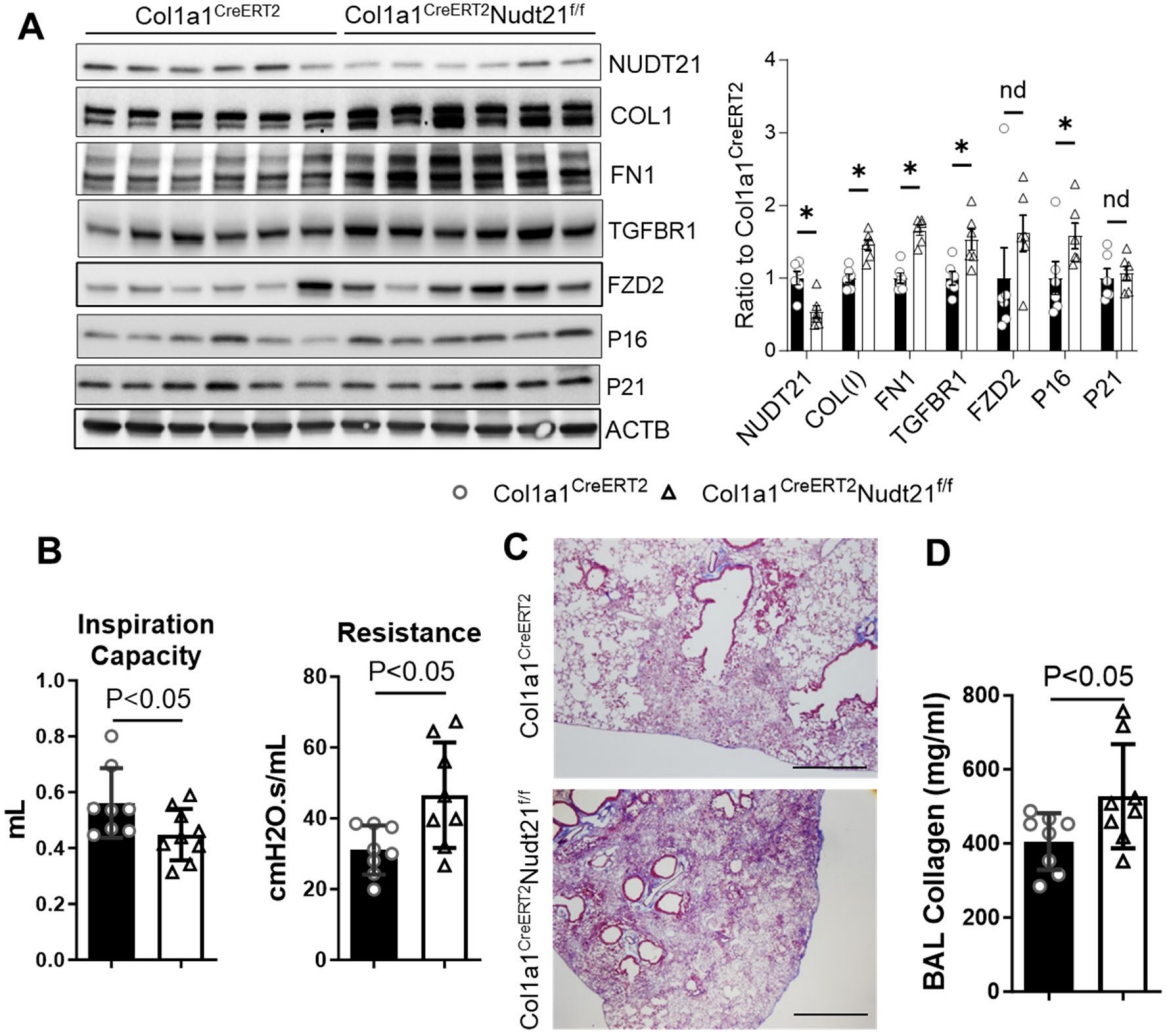
*Nudt21* fibroblast knockout (KO) exacerbates bleomycin‐induced pulmonary fibrosis in 18‐month‐old mice. Two‐month‐old *Col1a1* ^*creERT2*^ *‐Nudt21* ^*f*/f^ or control *Col1a1* ^*creERT2*^ mice were injected with ~75 mg tamoxifen/kg for five consecutive days each month until 13 months old. Starting 1 week after the last tamoxifen injection to induce pulmonary fibrosis, mice were i.p. injected with bleomycin (0.02 U/g) bi‐weekly for 4 weeks. Lungs were analyzed 28 days after bleomycin treatment. (A) Western blot (left) and densitometry (right) show NUDT21, COL1, FN1, TGFBR1, FZD2, P16, and P21 protein levels in control and *Col1a1* ^*creERT2*^ *‐Nudt21* ^*f/f*^ mice. (B) Pulmonary function parameters that have significant changes between the two groups were shown. (C) Representative Masson's Trichrome staining pictures from different groups. (D) Sircol assay showed soluble collagen levels in BAL. *p*‐value is calculated based on a two‐tailed Student *t*‐test. *N* = 6, scale bar = 400 μM.

To determine whether fibroblast NUDT21‐loss amplifies fibrosis in aging when combined with external fibrotic triggers, we performed experiments in aged animal models of fibrosis. For this purpose, we utilized 18‐month‐old bleomycin‐treated male *Col1a1* ^*creERT2*^ *‐Nudt21* ^*f/f*^ and *Col1a1* ^*creERT2*^ mice. A reduced bleomycin dose was selected due to the increased sensitivity of aged mice, which exhibited significantly greater weight loss (Figure S4A) and higher mortality (Figure S4B) compared to young mice at the standard dose of 0.035 U/g and are known to develop more severe lung fibrosis. Both groups were administered 0.02 U/g bleomycin via intraperitoneal bi‐weekly for 4 weeks. NUDT21 protein levels were decreased in the lungs of *Col1a1* ^*creERT2*^ *‐Nudt21* ^*f/f*^ mice compared to control *Col1a1* ^*creERT2*^ mice, suggesting successful NUDT21 knockdown (Figure 7A). Consistent with decreased NUDT21 downregulation, the previously identified NUDT21 target TGFBR1 was significantly upregulated, while another target, FZD2, showed a trend toward increased expression in *Col1a1* ^*creERT2*^‐Nudt21^f/f^ lungs (Figure 7A). The protein levels of fibrotic markers COL(I) and FN1 were elevated in *Nudt21* knockout mice (Figure 7A), suggesting increased fibrosis in *Col1a1* ^*creERT2*^ *‐Nudt21* ^*f/f*^ mice compared to control *Col1a1* ^*creERT2*^ mice. Markers of cellular senescence showed a selective increase, with elevated P16 but not P21 protein levels in the lungs of *Col1a1* ^*creERT2*^ *‐Nudt21* ^*f/f*^ mice (Figure 5A), further supporting the link between NUDT21 loss and senescence. Consistent with elevated COL(I) and FN1 levels, *Col1a1* ^*creERT2*^ *‐Nudt21* ^*f/f*^ mice exhibited reduced inspiratory capacity (IC) and increased resistance compared to controls (Figure 7B). Inspiratory capacity represents the maximum volume of air that can be inhaled after normal expiration. A reduction in IC suggests increased lung stiffness and reduced compliance in NUDT21 KO mice, likely due to fibrotic remodeling. Resistance measures the constriction of the lung. Elevated resistance in NUDT21 KO mice suggests increased airway constriction, which could be due to fibrotic remodeling and inflammation. Additionally, histological analysis with Masson's Trichrome staining revealed more extensive collagen deposition and airway remodeling in NUDT21 knockout mice (Figure 7C). These findings were corroborated by measurements of soluble collagen levels in BAL fluid, which were significantly elevated in *Col1a1* ^*creERT2*^ *‐Nudt21* ^*f/f*^ mice. Overall, these data indicate that fibroblast‐specific depletion of NUDT21 aggravates bleomycin‐induced pulmonary fibrosis in aged mice, highlighting the critical role of NUDT21 in mitigating fibrosis during aging.

## 4 Discussion

The central goal of this study is to investigate the role of NUDT21‐mediated APA in the context of IPF and aging. Our findings revealed that NUDT21, a critical regulator of APA, is significantly downregulated in aging lungs and fibroblasts. Additionally, NUDT21 downregulation is more pronounced in fibrotic lungs and fibroblasts, suggesting that reduced NUDT21 levels may contribute to aging‐associated diseases such as IPF. We also found that the loss of NUDT21 in fibroblasts is associated with increased phosphorylation of STAT3 and elevated expression of SASP factors (Graphical Abstract). Consistently, NUDT21 downregulation promotes fibroblast senescence and increases ROS production. Importantly, NUDT21 overexpression prevents STAT3 phosphorylation and the senescence phenotype in fibroblasts. Consistent with our in vitro findings, in vivo studies show that NUDT21 depletion in fibroblasts exacerbates bleomycin‐induced pulmonary fibrosis in mice, highlighting its critical role in modulating fibrotic responses. Collectively, these results establish that NUDT21 downregulation plays a critical role in the pathogenesis of IPF during aging by promoting fibroblast senescence and SASP release, positioning NUDT21 as a potential therapeutic target for addressing aging‐associated pulmonary fibrosis.

One of the hallmarks of IPF, an age‐related disease, is an increased number of senescent cells (Schafer et al. 2017; Wolters et al. 2014). Senescent cells, while not actively dividing, are metabolically active and produce a range of bioactive molecules, including pro‐inflammatory cytokines, chemokines, and matrix‐degrading enzymes. While much of the research has focused on epithelial cell senescence (Confalonieri et al. 2022; Parimon et al. 2023), studies have also demonstrated a significant increase in senescent fibroblasts in IPF lungs, driven by oxidative stress, environmental toxins, and telomere shortening (Pardo and Selman 2016; Waters et al. 2018; Alvarez et al. 2017; Lin and Xu 2020). Senescent fibroblasts show elevated levels of senescence‐associated β‐galactosidase (SA‐β‐gal), P16, P21, and P53. They also exhibit resistance to apoptosis and rapidly accumulate fibrotic markers, such as α‐smooth muscle actin (SMA) in primary cultures (Alvarez et al. 2017). Recent research has shown that senescent IPF lung fibroblasts promote the migration of alveolar epithelial cells, contributing to the fibrotic environment (Blokland et al. 2020). Moreover, senescent fibroblasts also show an increased release of SASP factors (Schafer et al. 2017; Alvarez et al. 2017; Ramos et al. 2001). These SASP factors include a wide range of pro‐inflammatory cytokines, chemokines, proteases, and growth factors crucial to a chronic inflammatory microenvironment. They not only perpetuate fibroblast senescence but also create a feedback loop that exacerbates tissue damage and fibrosis. Our findings suggest that NUDT21 is downregulated in senescent fibroblasts, and its depletion accelerates fibroblast senescence, creating a positive feedback loop that amplifies pro‐fibrotic signals. Given the detrimental role of fibroblast senescence in IPF, NUDT21 downregulation in senescent cells may contribute to the pathogenesis of fibrosis during aging. Consistently, our data demonstrated that NUDT21 depletion in fibroblasts increases collagen and fibronectin expression, contributing to excessive extracellular matrix deposition. Lung functional analysis revealed reduced IC and airway resistance in NUDT21 knockout mice. The reduction in IC indicates increased lung stiffness and reduced compliance, likely caused by fibrotic remodeling and excessive extracellular matrix deposition, which limit alveolar expansion. The elevated resistance suggests increased lung constriction, potentially driven by fibrotic remodeling or inflammation. These findings revealed a novel role of NUDT21 in pulmonary fibrosis pathogenesis during aging.

The mechanism for increased SASP expression in senescent fibroblasts is poorly defined, especially at the post‐transcriptional levels. A notable aspect of our findings is the connection between NUDT21 depletion and the upregulation of SASP factors in senescent fibroblasts through STAT3 activation. While STAT3 activation is well‐known for supporting tumor cell survival and growth (Grivennikov et al. 2009), recent studies have revealed enhanced STAT3 activation in senescent cells, underscoring the importance of the STAT3/senescence axis in various diseases. Extensive research on the JAK/STAT pathway in aging has revealed its critical role in regulating cytokine production as part of the SASP (Adnot et al. 2019), and inhibition of STAT3 has been shown to protect against cell senescence by reducing SASP factors (Waters et al. 2019). Consistently, IL6‐induced STAT3 activation promotes the senescence of human fibroblasts in association with increased ROS, DNA damage, and P53 accumulation (Kojima et al. 2012). Our RNA‐sequencing analysis revealed that several genes (e.g., IL6, EGFR, WNT5A, FZD2) involved in STAT3 signaling displayed 3′ UTR shortening upon NUDT21 depletion. This shortening may increase the stability and translation of these transcripts and ultimately promote STAT3 activation. Consistently, phosphorylated STAT3 (P‐STAT3 Y705) is elevated in NUDT21‐depleted fibroblasts. Inhibiting STAT3 signaling using either siRNAs to knock down expression of the key gene JAK2 or a STAT3 inhibitor STATTIC in NUDT21‐deficient fibroblasts significantly reduced the expression of several SASP factors, including IL6 and IL8, suggesting that STAT3 is a key upstream regulator of the senescence‐associated secretome. Overall, these findings indicate that NUDT21‐loss mediated STAT3 activation and SASP expression contribute to the chronic inflammatory state observed in aging or fibrotic lungs, highlighting the complex interplay between APA, SASP, and inflammatory signaling in IPF. Therefore, targeting NUDT21 or the APA machinery could serve as a novel therapeutic strategy for mitigating STAT3 signaling and chronic inflammation and fibrosis in IPF.

Several studies have highlighted the role of APA in cell senescence by controlling key aspects of mRNA stability and translation. For example, the downregulation of the splicing factor SRSF3 has been shown to promote cellular senescence via an APA‐dependent mechanism, in addition to its known function in alternative splicing (Shen et al. 2019). SRSF3 knockdown leads to 3′ UTR shortening of genes, including PTEN, PIAS1, and DNMT3A, thereby increasing their protein production and inducing senescence‐related phenotypes in both human and mouse cells. Similarly, in aged muscle stem cells, the accumulation of U1 snRNA, an APA factor, results in the expression of longer isoforms of CD47, which impairs cell proliferation (Porpiglia et al. 2022). In contrast, hematopoietic stem cells exiting quiescence exhibit a global shift toward shorter transcript isoforms (Sommerkamp et al. 2020). APA‐mediated 3′ UTR lengthening of the cancer‐associated gene CDC73 represses its expression and contributes to cancer cell senescence (Jia et al. 2018). Additionally, HuR, often repressed in senescent cells and elevated in proliferating cells, self‐regulates its expression by influencing APA site usage (Dai et al. 2012). Our studies uncovered a novel role for NUDT21‐mediated APA in fibroblast senescence. Our data indicate that NUDT21 downregulation induces a senescence‐like state in human lung fibroblasts. This is supported by increased expression of senescence‐associated markers, including COL1 and P53, along with components of the SASP. A significant elevation in β‐galactosidase activity, a hallmark of cellular senescence, further supports this phenotype. Additionally, we observed a reduction in lamin B1 and increased p‐H2A.X foci, indicative of DNA damage accumulation. Using the DCFDA/H2DCFDA Cellular ROS Assay Kit, we also detected a significantly higher proportion of ROS‐high fibroblasts in the NUDT21 knockdown group compared to controls, suggesting the presence of oxidative stress. Importantly, we found that OCR was significantly increased in NUDT21‐deficient fibroblasts. Although mitochondrial dysfunction is often associated with senescence, this finding is consistent with numerous studies showing that senescent or aging cells can exhibit elevated OCR and increased mitochondrial activity (Kim et al. 2018; Takebayashi et al. 2015; Kim et al. 2023). While some canonical markers such as P16 and CPEB family transcription factors were not significantly altered, this does not exclude the presence of senescence. Cellular senescence is a heterogeneous process, and marker expression can vary depending on the stimulus and cell type. Collectively, our results suggest that NUDT21 loss promotes a non‐canonical or partial senescence phenotype, defined by oxidative stress, DNA damage, SASP induction, metabolic reprogramming, and increased β‐galactosidase activity. These features may contribute to the pro‐fibrotic and pro‐inflammatory microenvironment observed in aging and fibrotic lung tissues.

In summary, our study identifies NUDT21‐mediated APA as a novel regulator of fibroblast senescence and lung fibrosis. Targeting senescent cells to treat idiopathic pulmonary fibrosis (IPF) is emerging as a promising area of research, given the well‐established link between cellular senescence and IPF progression. Recent studies have shown the potential of targeting senescent cells through senolytic drugs that eliminate senescent cells or therapies that modulate SASP factor secretion. Our data demonstrate that NUDT21 overexpression suppresses senescence markers and SASP factors in fibroblasts, further supporting its role as a key regulator of senescence. Particularly, our findings open new avenues for therapeutic intervention by targeting NUDT21 and the APA machinery to ameliorate fibroblast senescence and decelerate IPF progression. In addition to efforts to develop small molecules specifically targeting NUDT21 pathways, gene therapy strategies aimed at restoring NUDT21 expression could offer a novel therapeutic approach. Future studies will focus on identifying the upstream regulators of NUDT21 in aging and fibrosis and exploring its potential as a therapeutic target in human pulmonary fibrosis.

## Author Contributions

Tingting Mills and Zheng Chen designed the study. Jingjing Huang, Maria Jose Gacha‐Garay, Yu Wang, Hydia Puente and Tingting Mills performed the described experiments. Hari KrishnaYalamanchili conducted bioinformatics analyses. Scott D. Collum, Harry Karmouty‐Quintana and Rene A. Girard provided human lung and fibroblast samples and shared resources. Tingting Mills wrote the manuscript. Zheng Chen, Seung‐Hee Yoo, Maria Jose Gacha‐Garay, Eric J. Wagner, Harry Karmouty‐Quintana, Rahat Hussain, Jayeshkumar Patel and Manish Patel reviewed and revised the manuscript.

## Conflicts of Interest

The authors declare no conflicts of interest.

## Supporting information


Data S1.

## Data Availability

The data supporting the findings of this study are publicly available in the Gene Expression Omnibus (GEO) under accession number GSE137276 and can be freely downloaded.
