# Supplementary material for: Alternative Polyadenylation Contributes to Fibroblast Senescence in Pulmonary Fibrosis
Source: Aging Cell. 2025 Jul 30;24(10):e70179. doi: 10.1111/acel.70179 (PMC12507410; doi:10.1111/acel.70179)
Supplement: Supplementary file 1 — Data S1. [file ACEL-24-e70179-s001.docx]

**
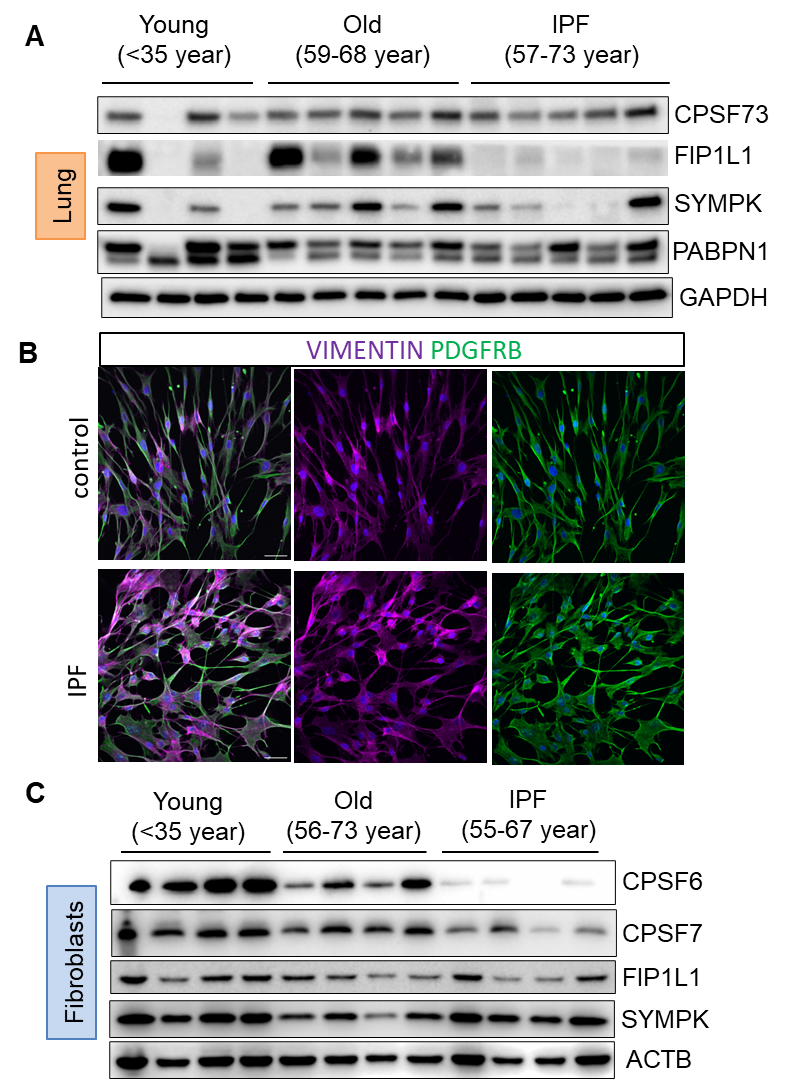
**

**Supplementary Figure 1. The expression of APA regulatory factors in aging and IPF lungs and fibroblasts.** (A) Western blot shows the expression levels of APA regulatory proteins (CPSF73, FIP1L1, SYMPK, PABPN1) in the lungs of young donors (< 35-year-old), aging donors (59-68-year-old), and IPF patients (57-73-year-old). (B) Fibroblasts isolated from normal or IPF donor lungs were stained with antibodies against Vimentin and plate-derived receptor beta (PDGFRB). (C) Western blot shows APA regulators (CPSF6, CPSF7, FIP1L1, and SYMPK) levels in lung fibroblasts isolated from young and old donors and IPF patients.


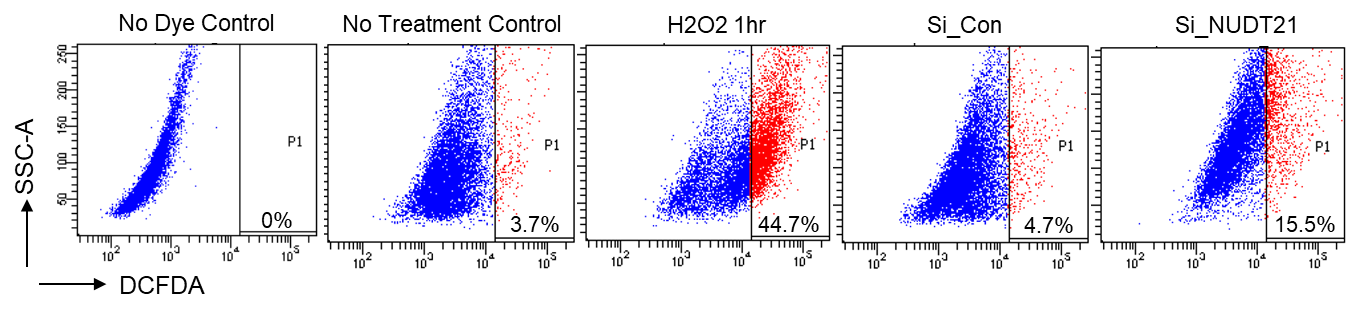


**Supplementary Figure 2. ROS in NUDT21 knockdown fibroblasts.** Normal human lung fibroblasts were transfected with either control siRNA or siRNA targeting NUDT21. Four days after the transfection, cells were stained using the DCFDA / H2DCFDA Cellular ROS Assay Kit according to the manufacturer’s instructions. Flow cytometry was performed to quantify ROS levels. An unstained negative control was included to determine background fluorescence. As a positive control, fibroblasts were treated with 800 μM hydrogen peroxide (H_2_O_2_) for 1 hour before staining.

**
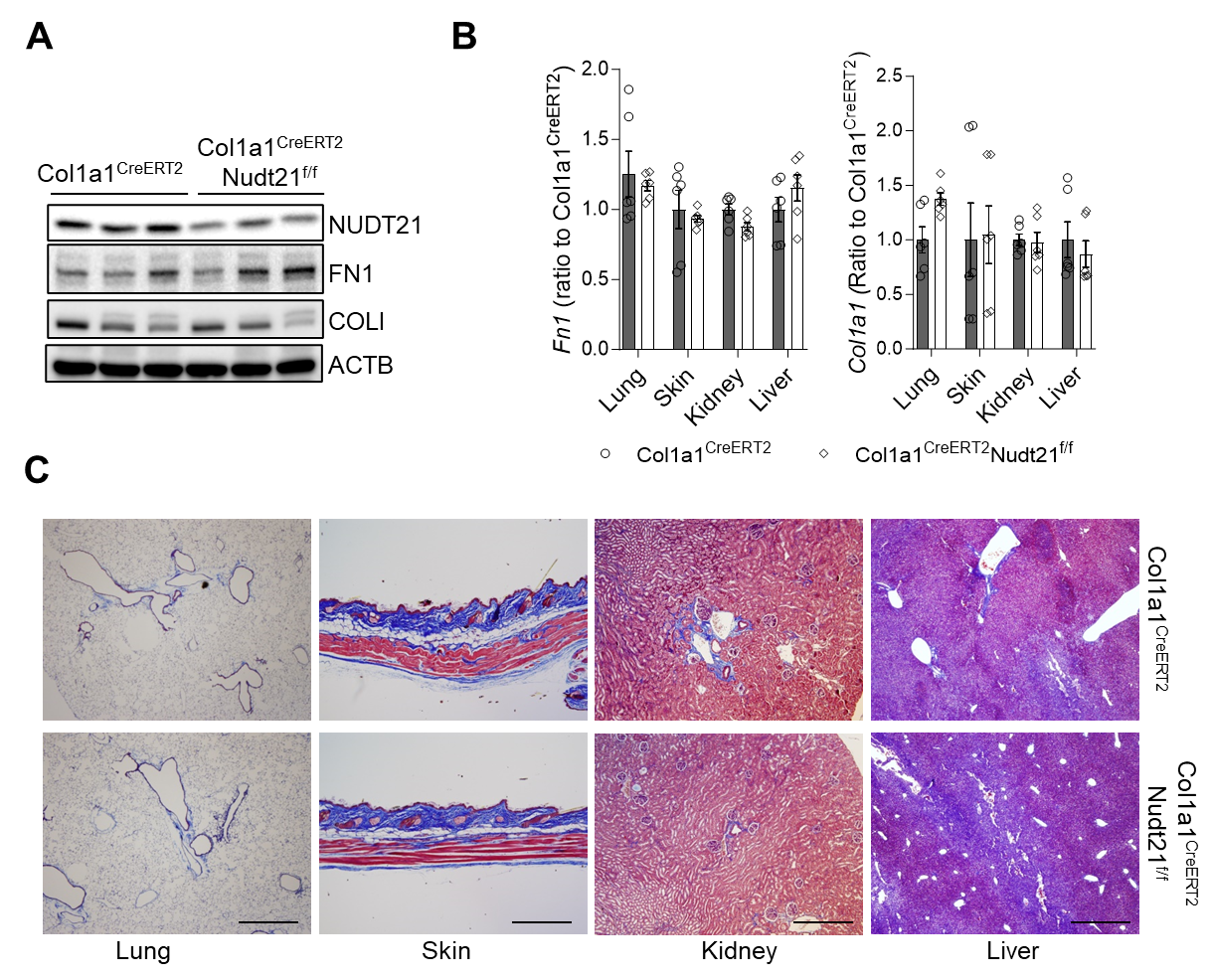
**

**Supplementary Figure 3. Aged Col1a1^cre/ERT2^-Nudt21^f/f^ mice do not develop spontaneous skin fibrosis.** Six- to Eight-week-old female *Col1a1^creERT2^-Nudt21^f^*^/f^ and control *Col1a1^creERT2^* mice were i.p. injected with 75 mg/day tamoxifen daily for 5 days and repeated every 4 weeks to ensure a stable deletion of Nutd21 in fibroblasts. The samples were collected at the age of 16 months for analysis. (A) Western blot of NUDT21, FN1 and COLI in the lungs of *Col1a1^creERT2^-Nudt21^f^*^/f^ mice compared to control *Col1a1^creERT2^*. (B) Real-time qRT-PCR showed the levels of *FN1* and *Col1a1* expression in the skin, lungs, kidneys and livers of *Col1a1^creERT2^-Nudt21^f^*^/f^ and control mice. (C) Masson’s Trichrome staining was carried out to visualize collagen levels in the lungs, skin, kidneys and liver. N=6, scale bar = 400 μM.


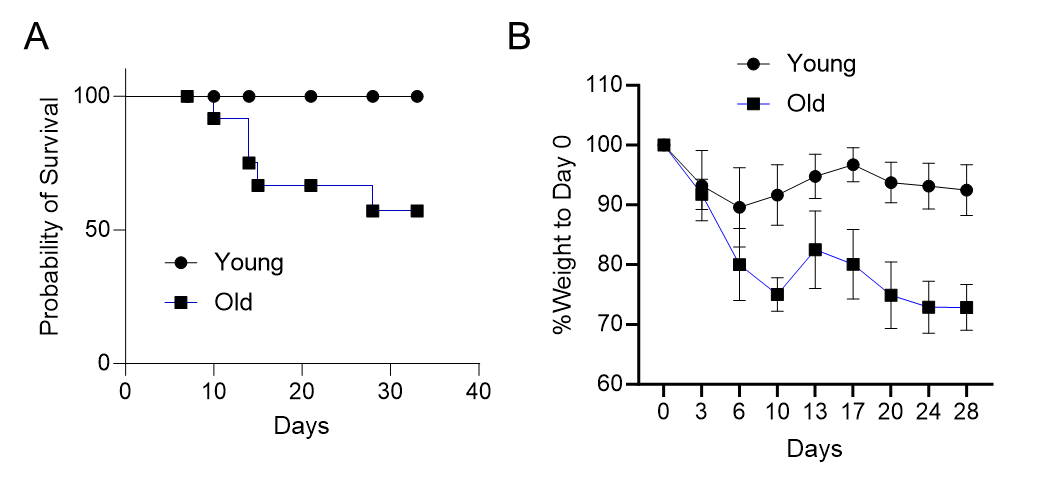


**Supplementary Figure 4. Survival curve and weight changes in bleomycin treated young and old mice.** Six- to eight-week-old (young) and 18-month-old (old) male C57BL/6 wild-type mice were intraperitoneally injected with bleomycin (0.035 U/g) twice per week for four weeks. (A) The survival curve shows 100% survival in young mice, while nearly 50% of old mice succumbed during the treatment period. (B) The percentage of body weight changes over time, indicating greater weight loss in old mice compared to young mice.

Supplementary Table 1: Demographic and clinical data for control and IPF-derived tissues.

| Figure 1A | | | | |
| --- | --- | --- | --- | --- |
| Sampels | Disease | Age | Gender | Race |
| Donor 1 | Normal | 22 | M | Caucasian |
| Donor 2 | Normal | 27 | M | African American |
| Donor 3 | Normal | 27 | M | African American |
| Donor 4 | Normal | 30 | M | Caucasian |
| Donor 5 | Normal | 59 | M | Hispanic |
| Donor 6 | Normal | 63 | M | Caucasian |
| Donor 7 | Normal | 60 | F | Caucasian |
| Donor 8 | Normal | 60 | F | Caucasian |
| Donor 9 | Normal | 68 | M | Hispanic |
| Donor 10 | IPF | 57 | M | Caucasian |
| Donor 11 | IPF | 65 | F | Others |
| Donor 12 | IPF | 63 | F | Caucasian |
| Donor 13 | IPF | 67 | F | Caucasian |
| Donor 14 | IPF | 73 | M | Caucasian |
| Figure 1B | | | | |
| Sampels | Disease | Age | Gender | Race |
| Donor 1 | Normal | 44 | M | Caucasian |
| Donor 2 | Normal | 58 |  | Other |
| Donor 3 | Normal | 65 | M | Caucasian |
| Donor 4 | Normal | 27 | M | African American |
| Donor 5 | Normal | 30 | M | Caucasian |
| Donor 6 | Normal | 25 | F | unknown |
| Donor 7 | Normal | 37 | M | Caucasian |
| Donor 8 | Normal | 33 | F | Caucasian |
| Donor 9 | Normal | 24 | F | Caucasian |
| Donor 11 | Normal | 39 | M | Hispanic |
| Donor 12 | Normal | 32 | M | Caucasian |
| Donor 13 | Normal | 60 | F | Caucasian |
| Donor 14 | Normal | 26 | M | Caucasian |
| Donor 15 | Normal | 25 | M | Hispanic |
| Donor 16 | Normal | 62 | F | Hispanic |
| Donor 17 | Normal | 21 | M | Caucasian |
| Donor 18 | Normal | 33 | M | Caucasian |
| Donor 19 | Normal | 43 | F | Other |
| Donor 20 | Normal | 56 | M | Hispanic |
| Donor 21 | Normal | 59 | M | Hispanic |
| Figure 1C, 1D | | | | |
| Sampels | Disease | Age | Gender | Race |
| Donor 1 | Normal | 44 | M | Caucasian |
| Donor 2 | Normal | 34 | F | African American |
| Donor 3 | Normal | 22 | M | Caucasian |
| Donor 4 | Normal | 27 | M | African American |
| Donor 5 | Normal | 37 | M | Caucasian |
| Donor 6 | Normal | 33 | F | Caucasian |
| Donor 7 | IPF | 63 | M | Caucasian |
| Donor 8 | IPF | 57 | M | Caucasian |
| Donor 9 | IPF | 63 | F | Caucasian |
| Donor 10 | IPF | 67 | F | Caucasian |
| Donor 11 | IPF | 43 | M | African American |
| Donor 12 | IPF | 68 | M | Unknown |
| Figure 1F | | | | |
| Sampels | Disease | Age | Gender | Race |
| Donor 1 | Normal | 21 | M | Caucasian |
| Donor 2 | Normal | 33 | M | Caucasian |
| Donor 3 | Normal | 29 | M | Caucasian |
| Donor 4 | Normal | 43 | F | Other |
| Donor 5 | Normal | 56 | M | Hispanic |
| Donor 6 | Normal | 73 | F | Caucasian |
| Donor 7 | Normal | 63 | M | Caucasian |
| Donor 8 | Normal | 60 | F | Caucasian |
| Donor 9 | IPF | 55 | M | African American |
| Donor 10 | IPF | 67 | M | Caucasian |
| Donor 11 | IPF | 52 | F | Caucasian |
| Donor 12 | IPF | 64 | F | Caucasian |

Supplementary Table 2:

| **Gene** | **Forward primer** | **Reverse Primer** |
| --- | --- | --- |
| Homo_NUDT21 | TGAAGTTGAAGGACTAAAACGCT | ACCAGTTACCAATGCAATCGTC |
| Mus_COL1A1 | GCTCCTCTTAGGGGCCACT | CCACGTCTCACCATTGGGG |
| Mus_FN1 | GCTCAGCAAATCGTGCAGC | CTAGGTAGGTCCGTTCCCACT |
| Mus_Homo_18S | GTAACCCGTTGAACCCCATT | CCATCCAATCGGTAGTAGCG |
| Homo_CSF2 | GGCCCCTTGACCATGATG | TCTGGGTTGCACAGGAAGTTT |
| Homo_IL6 | AATTCGGTACATCCTCGACGG | TTGGAAGGTTCAGGTTGTTTTCT |
| Homo_IL1b | ATGATGGCTTATTACAGTGGCAA | GTCGGAGATTCGTAGCTGGA |
| Homo_IL8 | CTTTCCACCCCAAATTTATCAAAG | CAGACAGAGCTCTCTTCCATCAGA |
| Homo_CXCL1 | ACTGCTGCTCCTGCTCCT | CGATGATTTTCTTAACTATGGG |
| Homo_EGFR | TTGCCGCAAAGTGTGTAACG | GTCACCCCTAAATGCCACCG |
| Homo_EGFR Long | GTAGCAGGCAGTGTGTTTTCC | TCTTAGTGCTTCTGCTGGGA |
| Homo_FZD2 | GTGCCATCCTATCTCAGCTACA | CTGCATGTCTACCAAGTACGTG |
| Homo_FZD2 Long | CTTTGCTGGTGTGAGAACTCC | ATGGAAGATGGCGAAAACTGAG |
| Homo_IFNAR1 | ATTTACACCATTTCGCAAAGCTC | TCCAAAGCCCACATAACACTATC |
| Homo_IFNAR1 Long | CTGGCTTCTCGTCTAGCAGT | TGGGTAATCCCCAGTAGCAA |
| Homo_IL6 Long | ATGGAAAGTGGCTATGCAGT | AGCCATTTATTTGAGGTAAGCCT |
| Homo_JAK2 | ATCCACCCAACCATGTCTTCC | ATTCCATGCCGATAGGCTCTG |
| Homo_JAK2 Long | CCTTTTTAGAGGGGAAATGAGGT | ATGACAACAACGAACAACCCC |
| Homo_STAM2 | GGAAAAAGCCACGAATGAGTACA | CAATCTTTCGCTCCATTAGGAGT |
| Homo_STAM2 Long | GAGGCATTATGTTGCTTGTGTG | AGGGGTGGCTACTGTTAAGTC |
| Homo_COL1A1 | GTGCGATGACGTGATCTGTGA | CGGTGGTTTCTTGGTCGGT |
| Homo_COL1A1 Long | GTGAGGGAGACAGACACCTG | GTGTTCTGGGGATTCAGGAG |
| Homo_ACTA1 | CATGTACGTTGCTATCCAGGC | CTCCTTAATGTCACGCACGAT |
